# Supplementary material for: Cyclosporin A as a Source for a Novel Insecticidal Product for Controlling Spodoptera frugiperda
Source: Toxins (Basel). 2022 Oct 21;14(10):721. doi: 10.3390/toxins14100721 (PMC9610628; doi:10.3390/toxins14100721)
Supplement: Supplementary file 1 [file toxins-14-00721-s001.zip › toxins-1912743-supplementary.pdf]

Supplementary Materials: Cyclosporin A as a Source for a Novel Insecticidal Product for Controlling *Spodoptera frugiperda*

Chengxian Sun, Shunjia Li, Kai Wang, Hongqiang Feng, Caihong Tian, Xiaoguang Liu, Xiang Li, Xinming Yin, Yanmei Wang, Jizhen Wei and Shiheng An

Supplementary methods and materials

Insect rearing

FAW individuals were reared at a temperature of 25 ± 1°C, relative humidity of 60 ± 10%, under a 16-h/8-h light/dark photoperiod at Henan Agricultural University. Eggs laid by female adults after mating were stored in sealed plastic bags with adequate moisture to hatch into larvae. The newly hatched larvae were fed fresh corn leaves in plastic boxes until they developed to the 3rd-instar stage. To prevent cannibalism among larvae, 3rd-instar larvae were reared individually with approximately 0.5 g of artificial diet (Liang et al., 1999) in 25 mL cups. The artificial diet was replaced daily until pupation. The sexes of newly-emerged adults were distinguished according to the external genitalia at the end of the abdomen. The adults were reared in cages with 10% sucrose solution for subsequent mating and reproduction. The female/male ratio was 1 : 1.2.

Calculation of expected mortality

We calculated the expected mortality for each CsA + other insecticide combination as follows:

$$\text{Expected mortality} = (1 - S_{CsA} \times S_X) \times 100\%$$

where S_{CsA} is the observed proportion of larvae that survived exposure to CsA, Sx is the observed proportion of larvae that survived exposure to the insecticide combined with CsA, and $S_{CsA} \times S_X$ is the proportion of larvae expected to survive a combination of CsA and another insecticide. The observed mortality was calculated by dividing the number of dead individuals by the total number of selected insects. If the observed and expected mortality were similar, the insecticides were considered to show independent toxic effects; if the expected mortality was significantly lower than the observed mortality, the combination of insecticides showed synergistic effects; and if the expected mortality was significantly higher than the observed mortality, the two insecticides showed antagonistic toxic effects .

Bioassay of sublethal CsA doses against FAW

Third-instar larvae were fed an artificial diet containing different doses of CsA, and all live larvae in each treatment (120 3rd-instar larvae of 3 replicates) were weighed every 2 days. At 7 days after CsA application, the numbers of larvae in different instar stages were recorded (3rd-instar larvae were recorded as dead). The larval development stages were distinguished by molting time. Larvae in the prepupal stage were observed every 12 h to determine the time of successful pupation. Thus, the development period was recorded from the 3rd-instar stage until successful pupation. Additionally, the numbers of larvae in different instar stages were recorded after 8 days of treatment (360 larvae of 3 replicates for each treatment).

For each treatment, pupation rates were calculated by dividing the number of pupae (including abnormal pupae) by the number of larvae (120 individuals for each replicate). Malformation rates were calculated by dividing the number of abnormal pupae by the total number of all pupae. Female and male pupae were distinguished according to their abdomen characteristics for analysis of the female/male ratio. Female and male pupae (including malformed pupae) were weighed 1 day after pupation. The pupal stage was defined as the number of days from pupae to successful emergence.

All adults received a 10% sucrose solution without CsA or DMSO every daily. Newly-emerged adults in each treatment group were transferred singly to a 25 mL cup. Eclosion rates were obtained by dividing the number of successfully emerged adults by the number of normal pupae. The adult period

(excluding mating) was defined as the number of days from successful adult emergence until death. At least 27 female adults of 3 replicates were used to investigate the sublethal effects of CsA on ovarian development. The number of mature eggs in ovaries (eggs between the ovipositor and pink parts of the oviducts; Fig. 5C) and ovarian length were recorded for 5-day-old virgin female adults. In the mating survey, vigorous female and male adults in each treatment (at least 19 pairs for each replicate) were selected on the 4th-day after emergence and placed singly in a 450 mL box covered with a piece of cotton gauze. After 1 day of mating, female adults with hard spermatophores were considered to have mated. To investigate the effects of CsA on FAW fecundity, a pair of 3-day-old adults were placed singly in a box, and the female was observed every morning; the date when the female began to lay eggs was recorded. Once the female began to lay eggs on the cotton gauze and box wall, the pair of adults was transferred to a new box covered with a piece of cotton gauze every day to facilitate egg counting; the eggs were collected into a sealed plastic bag with sufficient moisture. The hatching rate was analyzed according by the number of newly hatched larvae and number of eggs laid by females.
Larvae (8 days after CsA application), pupae (2 days after pupation) and adults (1 day after emergence) in the different treatments were observed using a camera (EOS R, Canon, Tokyo Japan). Ovaries were photographed using an anatomical microscope (M205 A; Leica, Welzlar, Germany).

Reference

Liang, G.; Tan, W.; Guo, Y. Improvement of artificial rearing technique of *Helicoverpa armigera*. *Plant Protection*, **1999**, 25, 15–17. https://doi.org/10.3969/j.issn.0529-1542.1999.02.006

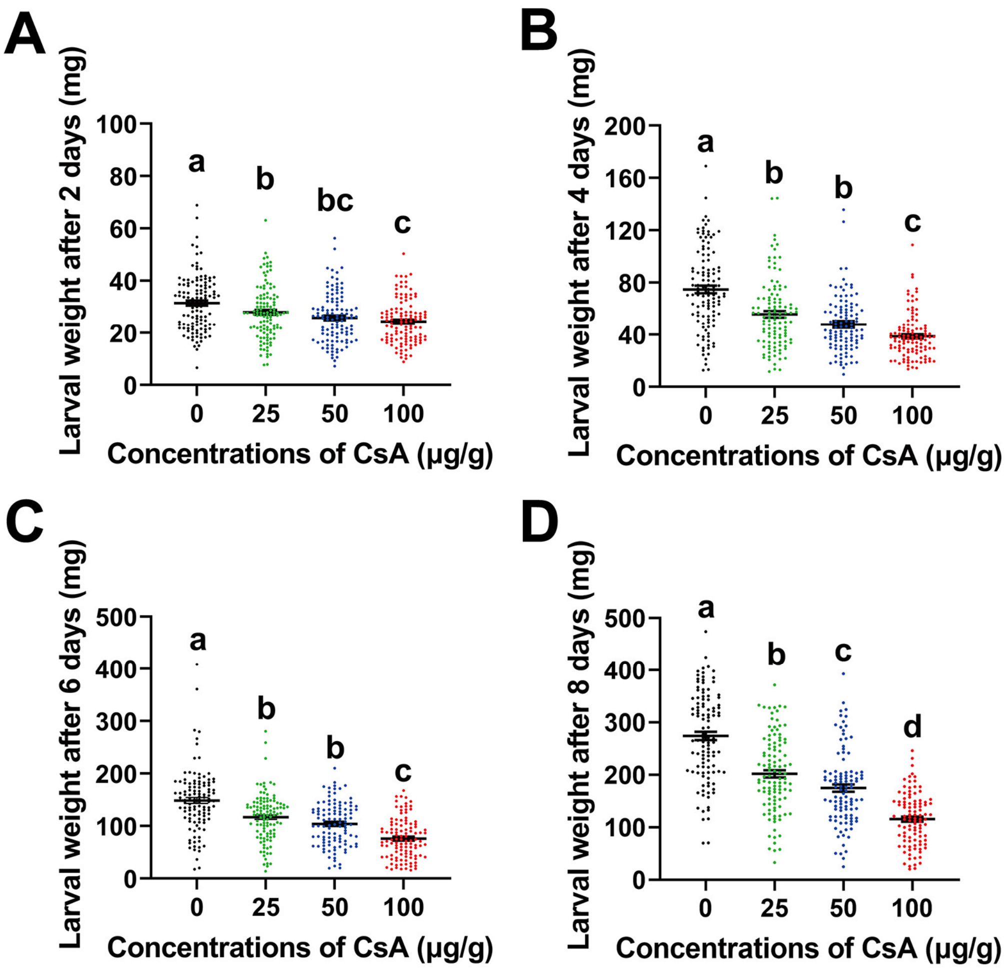

Figure S1. Negative effects of CsA on larval weights after 2 (A), 4 (B), 6 (C), and 8 days (D) of treatments. Data are means ± SE of three biological replicates. The lowercase letters upon error bars

displayed the significant differences at the level of $p < 0.05$, which were calculated by using ANOVA followed by Tukey test.

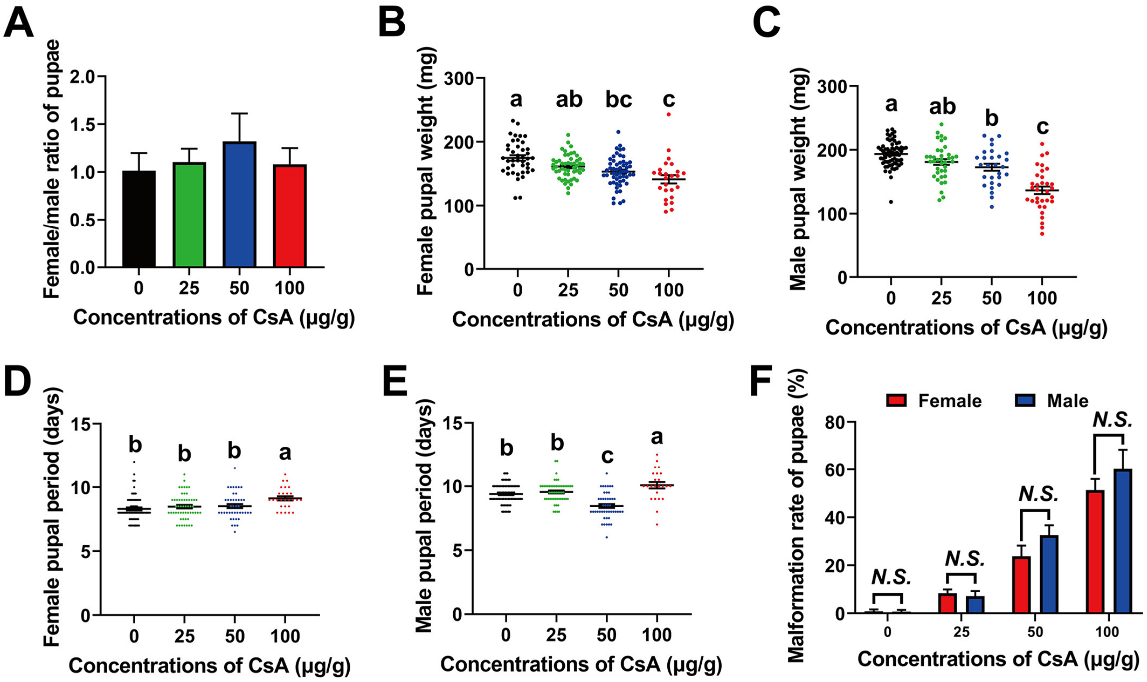

Figure S2. Effects of CsA on pupae. (**A**) Effects of CsA on the ratio of female/male pupae. (**B, C**) Fresh weights of female and male pupae treated with CsA. (**D, E**) Effects of CsA on pupal periods of females and males. (**F**) Malformation rates of female and male pupae caused by CsA. Data are means ± SE of more than three biological replicates. B–E: the lowercase letters upon error bars displayed the significant differences at the level of $p < 0.05$, which were calculated using ANOVA followed by Tukey test. F: "*N.S.*" means $p > 0.05$, and independent samples *t*-test was used for significant difference analysis between each pair of data.

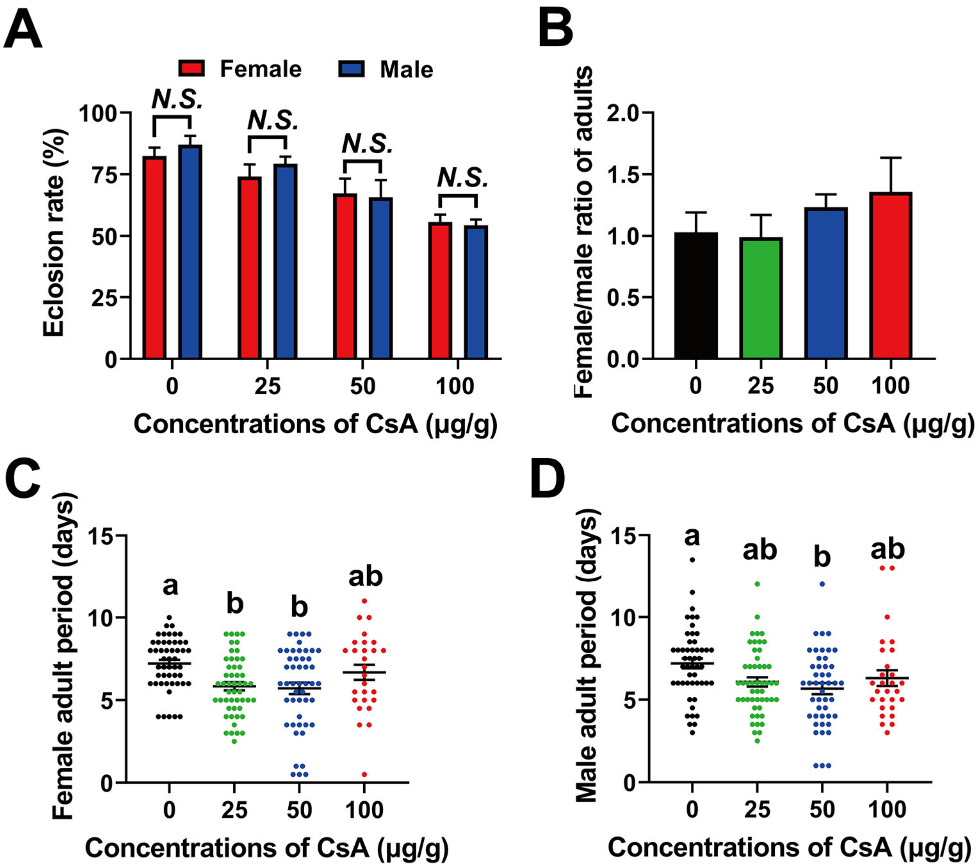

Figure S3. Effects of CsA on female and male adults. (**A**) The eclosion rates of female and male after different concentrations of CsA applications. (**B**) Female/male ratio of adults treated with CsA. (**C, D**) The changes of female and male periods caused by different concentrations of CsA. The error bars are means ± SE of more than three biological replicates. ANOVA followed by Tukey test at the level of $p < 0.05$ in SPSS 20 software was used for significant differences of multiple comparisons, and were marked with different lowercase letters. Student's *t*-test was used for significant differences of pairwise comparison, "*N.S.*" means $p > 0.05$.

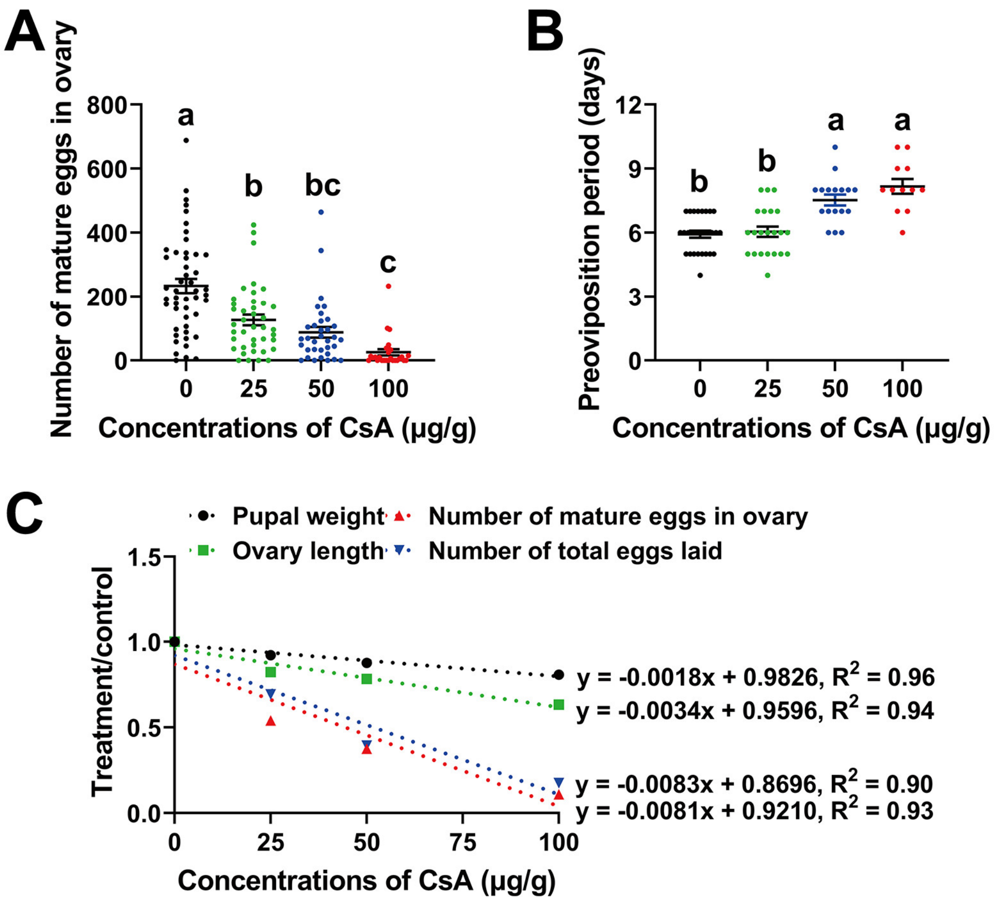

Figure S4. Negative effects of CsA on reproduction. (**A**) Effects of CsA on numbers of mature eggs in ovaries. (**B**) Preoviposition periods of female adults treated with different concentrations of CsA. (**C**) The change curves of pupal weight, numbers of mature eggs in ovaries, ovary lengths and numbers of eggs laid by adults caused by CsA. A, B: At least three biological replicates were used for calculating error bars displayed with means ± SE. The different lowercase letters marked upon error bars indicate significant differences analyzed by using ANOVA followed by Tukey test at the level of $p < 0.05$ level in SPSS 20 software. C: The curves of CsA concentrations and four physical signs were analyzed on Excel software (Microsoft Office 2016) by using data under 0, 25, 50, and 100 μg/g CsA divided by those of controls.
